# Our New York Letter

**Published:** 1891-07

**Authors:** Wm. L. Russell

**Affiliations:** 151 East 50th St.; New York


### Correspondence.

### OUR NEW YORK LETTER.

NEW YORK, June 15, 1891.

### PATHOLOGY OF ECLAMPSIA.

At the last meeting of the Academy, the subject discussed was the pathology of eclampsia and albuminuria of pregnancy. Those who expected to hear something new went home disappointed, and we are still ignorant of the pathology underlying a large proportion of these cases. Dr. Richard Van Santvoord read a paper. He stated that in most cases in which an autopsy had been made, pressure on the renal veins was found. There were, however, cases in which albuminuria appeared too early to be accounted for in that way, and not infrequently it disappeared before the end of gestation. Anæmia and œdema of the brain were believed to be the cause of eclampsia in some instances, and in others hydræmia and high arterial tension. In general, it might be said that two principal theories had been advanced, the reflex and the toxic. The former was based on the assumption that reflex conditions resulted in contraction of the arterioles of the brain. The toxic theory was based on the belief that the disturbances of digestion and of the functions of the liver, so frequent in pregnant women, resulted in an increase of toxic matters in the blood.

Dr. H. C. Coe said that no single theory could be satisfactory from the practical standpoint. In some cases there was a toxæmic condition. In some of these there was probably chronic diseases of the kidneys; sometimes a pyelitis from pressure on the ureters from an old exudation. Such cases were very difficult of discovery. It was important to watch the quantity of urine passed. This was now done at the Maternity Hospital weekly.

Prof. Lusk believed that the objections against the renal origin of eclampsia had never been removed. The kidneys were occasionally found normal, and patients with well-marked kidney disease frequently escaped eclampsia. Anæmia could not be regarded as a cause alone, nor could toxæmia alone. Something else must be added to either. Three classes of cases were met with in practice; those in which there was a sudden stoppage of urine probably from reflex causes, with contraction of the uterus and arterioles; those in which there was arrest of urinary secretion, with disturbance of the reflex centre in the medulla followed by contraction of all the arterioles in the body, including those of the brain; those in which the condition just mentioned existed with anæmia of the great centres, but without kidney trouble. The treatment consisted in emptying the uterus as soon as possible, all other measures being temporary only.

The President, Dr. Loomis, said that though he had not seen many cases of eclampsia for a number of years, he had long ago observed that the danger of a pregnant woman depended on the amount of arterial tension present. A difference in tension was noticeable in different individuals in many conditions, as after a hearty meal or the use of stimulants.

In closing the discussion Dr. Van Santvoord said that he favored the toxic theory. There was something behind the renal trouble, and the high arterial tension which had been so generally noticed. Toxæmia was probably at the root of the trouble. The elimination of urea had not been sufficiently inquired into. It was very easy to determine the quantity, and the fact that it was found diminished in the cases studied by him indicated that it might be a matter of some importance.

### DEEP URETHRAL CATARRH.

In a paper read at a recent meeting of the Academy, Dr. E. Keyes gave some suggestions for the treatment of deep urethral catarrh. He said that gleet was now recognized to be nothing more than a symptom, very often of a catarrh deep down in the urethra. Even when stricture was discovered and cured the discharge did not always cease, and it was always wise to warn the patient of that possibility. Usually gleet was a small affair, the discharge being very slight. When posterior urethral catarrh was present, however, it could be demonstrated that there was much more pus posterior to the bulbo-membranous juncture than the discharge would indicate. This was accomplished by washing out the anterior urethra by means of a soft catheter, and then by milking the posterior urethra by firm manipulation in the perineum; if the urine were then passed in two portions, the first would be found to contain much pus, while the second was also turbid. When the prostate or seminal vesicles were involved in the inflammation, the milking process, by means of the finger in the rectum, would reveal the true state of things.

In regard to treatment it could be stated that some cases recovered without local measures, under rest, alkalies, and balsams. Treatment of the anterior urethra had also a good effect sometimes. Many more, however, could be cured only by means of medication applied directly to the deep urethra. This could best be done by means of a proper syringe (Keyes' syringe). There was no danger of inducing cystitis or epididymitis if the instrument were not inserted too far. The tip should stop at the membranous portions of the urethra. The solutions used by him were of nitrate of silver and sulphate of copper, one to ten grains to an ounce. He had used many other substances, but had come to rely on these.

Dr. Samuel Alexander, in discussing Dr. Keyes' remarks, said that he never regarded a gleet as a small affair, for it sometimes produced poisonous symptoms. He also used the deep injections of silver and copper, ten or fifteen drops of either being enough to inject. Occasionally the discharge was increased, and sometimes a solution, as weak as four grains to an ounce, caused pain in the bladder. The endoscope was very useful in diagnosing these deep urethral catarrhs, and he thought it ought to be used. There was a tendency to throw internal treatment over ; this was a mistake, however, for it was often very useful.

Dr. G. E. Brewer had used Keyes' method for five years, and had found that many aggravated cases could be cured by the deep injections of nitrate of silver. He had seen cases in which there was bloody urine, and symptoms indicating stone in the bladder relieved in two weeks by this method.

Dr. F. A. Sturgis was also in favor of the Keyes' treatment. He injected but three to five minims of a very strong solution of silver nitrate. He had also found glycerole of tannin, sulphate of zinc, and acetate of zinc of service. He had used a silver solution as strong as thirty and even fifty grains to an ounce. The sulphate of zinc could be used in ointment with vaseline, by pushing it through the tube of the endoscope directly to the diseased point. This was much better than using a suppository, which contained too much white wax. In spite of all treatment some cases could not be cured.

TREATMENT OF GONORRHŒA.

This subject was taken up in the Genito-Urinary Section of the Academy on the last night of meeting. Dr. G. E. Brewer read a paper in which he warmly recommended the use of retrojections of solutions of bichloride of mercury, of varying strength. The method employed by him was to use retrojections of a solution of the strength of from 1 to 16,000 to 1 to 50,000 twice daily. When the acute stage had passed bismuth and glycerine were used. He had treated cases in this way for five years, and had treated by this and other methods about eleven hundred cases. The conclusion at which he had arrived was that the bichloride reduced pain and inflammation, and the liability to complications more satisfactorily than any other remedy. So far as cure was concerned, however, he had discovered that it was uncertain with any treatment.

Dr. Keyes said that he had also come to rely on the bichloride. It was least useful when a posterior urethral catarrh had been lit up into activity anew by a debauch. One difficulty which he had encountered in treating gonorrhœa was the cumbersomeness of the apparatus ordinarily recommended. He had lately overcome this by the use of a small soft rubber bulb, known as Tiernann's Universal Injector. It held about two ounces. He was accustomed to order one grain of bichloride of mercury, with ten grains of sulphate of potash or other inert substance, divided into ten powders. One of these was to be added to an ordinary tumblerful of water just hot enough to hold the finger in by exerting some will power. It dissolved instantly. The bulb was then filled and the beak introduced into the meatus. By gentle pressure the urethra should be filled until it felt tense ; then the bulb should be withdrawn, gentle pressure being continued until the solution welled up around the beak. The urethra should then be kept full until the solution in the tumbler was used up, pressure being made to prevent the escape of the contents, at each renewal of the quantity in the bulb. When the purulent stage had passed, astringents should be used. By this plan of treatment he had found that the discharge could usually be controlled in ten days or two weeks. It was the best with which he was familiar.

Dr. R. W. Taylor believed that it was the hot water which was principally accountable for the success of the treatment referred to. The parasitic treatment of gonorrhœa had been misleading to many minds, and often resulted in forgetfulness, on the part of those who used it, of the simple fact that there existed an intense inflammatory condition which should be treated on the same principles as other inflammations situated elsewhere. He thought that a solution of 1 to 50,000 was inert so far as the bichlorde was concerned. The effects were produced by the hot water. A few drops of lead water and laudanum might be added. A two or three per cent. solution of boracic acid was also a satisfactory addition. He thought that there was danger with Keyes' bulb of distending the urethra too much and causing rupture of the mucous membrane. He did not think that deep injections ought to be given as a matter of routine.

Dr. Keyes added that the sensations of the patient indicated that a solution of bichloride 1 to 50,000 was not altogether inert. He usually increased the strength to one-sixth of a grain to the glass of water, and the stronger solutions frequently caused much pain.

In closing the discussion Dr. Brewer stated that he had tried hot water alone, with astringents, and with boric acid, but had found none of these so useful as the bichloride. It had a marked effect on the discharge. Solution of the strength of 1 to 30,000 sometimes caused burning, and in one instance a solution of 1 to 40,000 caused so much pain as to necessitate the use of cocaine. Sir Joseph Lister had found that a solution of 1 to 50,000 destroyed micro-organisms, while one of 1 to 200,000 prevented this multiplication. It was true that the retro-injection apparatus was clumsy, so he was accustomed to have his patients come to his office twice daily and have the urethra washed out. Where that was impracticable, a No. 18 soft rubber catheter and an Alpha siphon answered every purpose and could be used very conveniently.

Dr. Taylor again expressed doubts concerning the value of germicides, on account of the depth into the mucous membrane at which the micro-organisms were found. To this Dr. Brewer replied that although the germs penetrated deeply they developed near the surface. Dr. Taylor then suggested that the treatment was liable to provoke posterior urethritis. Dr. Brewer did not think so, for any plan of treatment which shortened the duration of the anterior inflammation lessened the chances of air invasion of the posterior portion of the canal.

WM. L. RUSSELL.

151 *East 50th St.*

---

VACCINATION.—Before the vaccination by Jenner was adopted, smallpox was one of the most formidable scourges of the human race, causing a mortality of 10 per cent. Since its general adoption it is less than 1 per cent. During the prevalence of smallpox in Prussia, from 1857 to 1861, nearly eight thousand among the civil population died. The mortality in the army, where vaccination was rigidly enforced, was practically *nil*.

During the period of thirty years just before the introduction of vaccine in the province of Trieste, the deaths from smallpox alone were 14,000 per 1,000,000 of inhabitants, and only 182 during the period of two years which followed the practice of vaccination.—*Weekly Medical Review.*

